# Formulation and In Vitro evaluation of pH sensitive oil entrapped polymeric blended gellan gum buoyant beads of clarithromycin

**Published:** 2010

**Authors:** G. Tripathi, S. Singh

**Affiliations:** Industrial Pharmaceutics Laboratory, Saroj Institute of Technology & Management, Lucknow-226001, India

**Keywords:** pH sensitive drug delivery system, Hydrophilic polymer, Buoyancy, Gastric retention

## Abstract

**Background and the purpose of the study:**

A gastroretentive pH sensitive system has been a frontier approach to release the drug in controlled manner in stomach and duodenum. The aim of this study was to develop buoyant beads of gellan based, wherein, the oil was entrapped, blended with hydroxypropyl methyl cellulose or carbopol 934 in order to evaluate its potential for targeted sustained delivery of clarithromycin in the gastric region.

**Methods:**

Buoyant beads of gellan was developed by inotropic gelation technique using calcium carbonate as gas forming agent and the drug polymer dispersion was emulsified with mineral oil. The oil was entrapped and blended with hydroxypropyl methyl cellulose or carbopol 934. The developed beads were evaluated in terms of diameter,% floating, encapsulation efficiency, In vitro drug release, In vivo gastric residence efficacy and clarithromycine concentration in the mucosa of the experimental animal model.

**Results:**

The scanning electron microscope photograph indicated that the prepared beads were spherical in shape and buoyancy, encapsulation efficiency and drug content obtained from all batches were satisfactory. Particle size and percentage buoyancy of the gel beads increased by raising the concentration of calcium carbonate. The formulation exhibited sustained release profile and was best fitted in the Peppas model with *n<*0.45. Subsequent coating of microbeads exhibited zero-order sustained pattern of the drug release up to 8 hrs. Batch B_4_ showed comparatively better residence and the drug concentration in the gastric mucosa of the treated animals.

**Conclusion:**

The result provides evidence that the prepared optimized formulation may be used effectively for pH sensitive gastric targeted antibiotic such as clarithromycin.

## INTRODUCTION

Oral delivery systems that can precisely control the release rate of target drug in a specific site of gastro-intestine (GI) have an enormous impact on the health care system. Gastroretentive drug delivery system is an approach to prolong gastric residence time and appropriate for drugs which should be locally active in the gastric mucosa in the stomach, in particular antibiotics administration for *Helicobacter pylori* (*H. pylori*) eradication in the treatment of peptic ulcer and other diseases (1).

*Helicobacter pylori (H. pylori)* is a small, spiral, microaerophilic, gram-negative bacteria which has been recognized to be associated with gastritis and duodenal ulcers. The microorganism has also been reported to be involved in the pathogenesis of other diseases, such as chronic atrophic gastritis, adenocarcinoma of the body or antrum of the stomach, gastro-esophageal reflux diseases, peptic esophagi and etc (2, 3). The bacteria penetrates to the gastric mucous layer and fix itself to various phospholipids and glycolipids in the mucus gel. Therefore, access of antimicrobial drugs to the site is restricted from both the lumen of the stomach and the gastric blood supply. Gastroretentive systems have gained considerable attention due to their ability to adhere to the mucus layer, as well as to release the drug in a sustained manner in the mucosa of the stomach (4). The extended release of the drug can maintain a higher antibiotic concentration in the gastric region where *H. pylori* exists and thereby improve the therapeutic efficacy. The bioadhesive drug delivery system can plug and seal the infected and inflamed mucosal cell lines (5). Preparation and evaluation of stomach-specific controlled release mucoadhesive drug delivery system containing acid- soluble drug amoxicillin trihydrate has been reported (6). Also, gellan gum based floating beads containing clarithromycin have been prepared by iontotropic gelation method for stomach-specific drug delivery against *H.pylori* and have shown good antibacterial activities against isolated *H. pylori* strains (7).

Clarithromycin (Cl) is a broad spectrum macrolide antibacterial agent that is primarily bacteriostatic and its mechanism of action is binding to the 50 S ribosomal subunit of susceptible organism and inhibiting protein synthesis (8).

The primary objective of the present work was to develop a consistent formulation of Cl that enjoys all the advantages of a floating single unit dosage form but at the same time being devoided of disadvantages of single unit dosage forms, like sticking or being obstructed in the gastrointestinal tract.

## MATERIAL AND METHODS

### Materials

Clarithromycin was obtained as gift sample from Ranbaxy Laboratories Ltd, Gurgoan, India. Gellan gum was purchased from Sigma-Aldrich Chemicals Ltd, New Delhi, India. Carbopol 934P and Hydroxypropyl methylcellulose K4M were obtained as a gift samples from, Ranboxy laboratory Devash, India. Calcium chloride and ethyl cellulose were obtained from S.D. Fine Chem. India. Light mineral oil was obtained from the Central Drug House, India. All other ingredients, reagents and solvents were of analytical grades.

### Methods

#### Preparation of polymeric blend oil entrapped bead

Oil entrapped polymeric blend gel bead of clarithromycin was prepared by ionic gelation method. Aqueous solution of gellan gum (1.5-2.0% w/v) was prepared using de-ionized water and heating at 70°C. The gellan gum solution below 35°C was successively dispersed into the slurry of 0.5 -1.0% w/v of HPMC or carbopol 934P in order to prepare specific ratio of blended polymeric dispersion (Table 1) with continue stirriring for 20 min. The drug (0.54% w/v) and calcium carbonate (0.55-1.5% w/w) were dispersed uniformly into 20 ml of the polymeric blended mixture with continuous stirring until a uniform dispersion was obtained. The mixture was emulsified with 05-15% w/v of light mineral oil using Silverson emulsifier (Hicon, L5M-4, India) with continuous stirring at 500 rpm for 5 min.

**Table 1:** Composition of the drug loaded polymeric blended gellan gum bead

Formulation		Drug	Gum	Oil	Calcium carbonate
				
(COB)	(HOB)	(% w/v)	(% w/w)	(% w/v)	(% w/v)
B_1_	N_1_	0.54	1.5:1.0	05	0.55
B_2_	N_2_	0.54	1.5:1.0	10	0.55
B_3_	N_3_	0.54	1.5:1.0	15	0.55
B_4_	N_4_	0.54	1.5:1.0	05	1.5
B_5_	N_5_	0.54	2.0:0.5	05	0.55
B_6_	N_6_	0.54	2.0:0.5	10	0.55
B_7_	N_7_	0.54	2.0:0.5	15	0.55
B_8_	N_8_	0.54	2.0:0.5	05	1.5

COB=gellan: carbopol blended formulation

HOB=gellan: hydroxypropyl methylcellulose (HPMC) blended formulation

Gum=gellan: carbopol /HPMC

Oil=mineral oil

The resulting drug loaded emulsions was added through a 21G syringe needle into 100 ml of 0.45 mol ml^−1^of calcium chloride (CaCl_2_) solution. After 5 min of curing time, the formed beads were washed with distilled water, collected and dried at 40°C for 6 hrs.

#### Coating of gel beads

Formulated microgel beads were selected for optimization in order to modify drug release pattern further. The coating parameters were 5-10% (w/v) ethylcellulose (EC) solution in acetone and coating times was fixed (5- 10 min). Gel beads (2 gr) were placed in a fluidized bed dryer (TG 100, Retsch, Germany) and the coating solution was sprayed on the fluidized beds using a spray gun for a period of 10 min at room temperature at an air inlet speed of 220 ms^-1^. The beads were dried at room temperature for a period of 24 hrs until all solvent was evaporated, leaving a film of EC coat on the gel beads (Table 2).

**Table 2:** Independent variables of the formulation bead coated with ethyl cellulose.

Formulation (code)	EC concentration (% w/v)	Time of coating (min)	% drug release t_480 (min.)_^a^	R^2^
B_14_	5	5	70±1.5	0.9424
B_24_	5	10	80±1.6	0.9745
B_34_	10	5	72±1.7	0.9313
B_44_	10	10	69±1.2	0.9232

EC=ethyl cellulose

R2=correlation coefficient derived from zero order drug release kinetics.

B_14_, B24, B34 and B_44_=ethylcellulose coated optimized formulation of batch B

a =Mean±SD (n=3)

#### Morphology and particle size

The external and internal morphology of microgel beads were studied by scanning electron microscopy (SEM). Particle size of the prepared beads were determined using an optical microscope (Model BH-2, Olympus, Japan) fitted with a stage and an ocular micrometer. Mean diameter was calculated by measurement of diameter of 20 dried beads.

#### In vitro floating study

In vitro floating study was performed using a USP-24 dissolution apparatus II containing 900 ml of simulated gastric fluid (SGF) of pH 1.2. The medium temperature was kept at 37±0.5 °C. The floating beads (1.0 gr beads) were soaked in the dissolution medium and the medium was agitated with a paddle at 50 rpm. After agitation, the beads that floated on the surface of the medium and those that settled down at bottom of the flask were recovered separately. The percentage of floating was measured by visual observation (9)

#### Encapsulation efficiency and drug content

Accurately weighed (100 mg) grounded powder of beads was soaked in 100 ml phosphate buffer (pH 7.5) and allowed to disintegrates completely for 4 hrs (9). The resulting dispersion was sonicated using a probe sonicator (UP 400 s, Dr. Hielscher GmbH, Germany) for 30 min and then filtered through a 0.45 µm filter. The polymeric debris was washed twice with fresh phosphate buffer to extract any adhered drug and drug content was determined spectrophotometrically at 353 nm against constructed calibration curve. The drug content (DC) was calculated according to Eq 1.

1 DC%=(amount of drug in beads/amount of beads)×100

The encapsulation efficiency (EE) was calculated by Eq 2.

2 EE(%)=(C/T)×100

Where C is the calculated drug content and T is the theoretical drug content.

#### Gastric residence efficacy of microbeads

Gastric residence efficacy was evaluated by the method of Zheng et al. (10) with slight modification. The protocol of the study was approved by the animal ethical committee of the department. Albino rats selected for the study were fasted for 8 hrs and then divided into two groups of three animals.

Each groups was pre-treated by an intraperitoneal injection of omeprazole at the dose of 15 mg/Kg to suppress gastric acid secretion. After one hour of the administration of omeprazole, each group was given a single oral dose of 500 beads (batch B_4_ orbatch N_5_) in aqueous suspension. After 1, 4, and 8 hrs, the rats were scarified by cervical dislocation and the stomachs were removed. The microbeads that retained in the stomach were counted and the percentage of the remaining beads was calculated.

#### Evaluation of concentration of the drug in gastric mucosa

Albino rats were fasted for 8 hrs and then divided into three groups of three animals. The protocol of the study was approved by the animal ethical committee of the department. The animals were treated by an intraperitoneal injection of omeprazole at the dose of 15 mg/Kg to suppress gastric acid secretion. After one hour of omeprazole treatment, one group was treated with Cl plain and other groups were treated with formulation batches B4 and N5 at the equivalent dose of 40 mg/ Kg. The rats were sacrificed at 1, 3, 6 hrs after administration, the stomachs of the rates were removed and opened along the great curvature, residues in the stomachs were removed carefully, and the stomachs were gently rinsed in 20 ml of distilled water and spread on a glass slide, and the top layer was separated from the muscular layers (11). The removed mucosa was mixed with phosphate buffer pH of 7.4 in a glass tissue glinder and homogenate was centrifuged in a refrigerated ultracentrifuge at 3500 rpm for 5 min. The supernatant was removed and filtered through0.45 µm filter. The amount of Cl contained in sample was measured by spectrophotometric method at 353 nm.

#### In vitro drug release

In vitro dissolution studies were performed for all formulation gel beads using USP 24 dissolution test apparatus II with a basket type. An accurately weighed 50 mg amount of the beads were taken in to 900 ml dissolution medium of the simulated gastric fluid ( fasting state condition, pH of 1.2,) or phthalate buffer solution, (fed state condition, pH of 3.4) and maintained at a temperature of 37°C±0.5°C and stirred at a speed of 50 rpm. Sample aliquots (10 ml) were withdrawn at 0.5, 1.0, 2.0, 3.0, 4.0, 5.0, 6.0, 7.0 and 8.0 hrs and volumes were replaced with an equivalent amount of the dissolution medium. The collected samples were filtered and analyzed at 353 nm using a UV-visible spectrophotometer. Additionally, an experimental batch F(fed state) and batch FF(empty state) containing 10 mg of Cl and lactose (q.s.) filled in a capsule (# 2) was used as a reference formulation.

#### Statistical analyses

The experimental results are expressed as mean±SD. Statistical evaluation of data was performed using an analysis of variance (ANOVA). The evaluation data was used to assess the significance of differences. Statistically significant difference between the means of batches were defined as P<0.05.

## RESULTS AND DISCUSSION

### 

#### Morphology and Particle size

**Figure 1:**
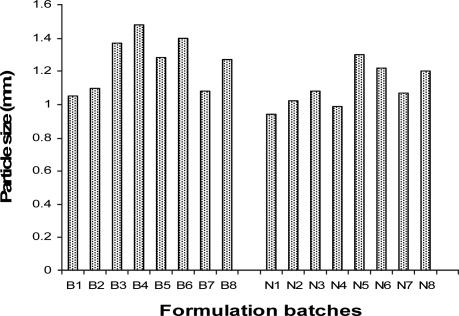
Mean particle size distribution pattern of different batches of formulations Hydroxypropyl Methylcellulose (HPMC) Blended Formulation (HOB) and Carbopol Blended Formulation (COB).

Spherical gel beads were formed instantaneously when emulsion was dropped into CaCl2 solutions. Gelation occurred due to intermolecular cross- linking between the divalent calcium ions (Ca+2) and the negatively charged carboxyl groups of gellan gum. SEM of Cl loaded oil entrapped blended with carbopol 934P (formulation COB) beads were white, translucent and rigid and HPMC blended (formulation HOB) beads were found to be spherical with smooth surfaces. The diameter of COB formulation varied between 1.05±0.8 mm. to 1.48±0.3 mm. while that of HOB was 0.94±0.4 mm. to 1.30±0.5 mm. Size distribution pattern of micro beads of both formulations showed significance differences (Fig 1). The diameter of the beads increased significantly (*p<*0.05) as polymer concentration increased; which viscosity of the polymeric dispersion, eventually leading to the formation of bigger beads. Larger size beads were also formed as the concentration of calcium carbonate (CaCO_3_) increased. This may be due to excess Ca+2 causing all possible cross-linking sites in the polymer to be fully utilized, resulting in large but weaker and flexible gel beads.

#### In vitro floating study

The formulations batch B_4_ and batch N_5_ were found to have good floating ability (92±1.2% and 89±1.3% respectively). The floating ability of the formulation mainly depends on CaCO_3_ and blended polymer ratio. Similar finding have been reported by Rajinikanth and Mishra (7) on evaluation of gellan bead of clarithromycin. By increasing CaCO_3_ concentration, floating percentage of the micro gel beads increased. The increase in amount of Ca+2 and consequently the amount of the evolved carbon dioxide gas (CO_2_), are entrapped in the gel network of the formulation, cause the gel rises to the surface of the dissolution medium (in vitro) or the stomach (12).

#### Encapsulation efficiency and drug content

Encapsulation of the drug was found to be consistently higher in the prepared formulations of COB (75±0.7-93± 0.4%) and HOB (70±0.5- 86±0.6%). No significant (P>0.05) effect was. observed for CaCO concentration on encapsulation efficiency of the prepared gel bead. The drug content of COB formulation was found to be in the range of 58±1.4 to 78±1.6% w/w. HOB formulation was found to be in the range of 44±1.6 to 68±1.8% w/w. No significant (P>0.05) effect was observed with CaCO_3_ and CaCl_2_ on drug content of beads.

#### Gastric residence efficacy of microbeads

**Figure 2:**
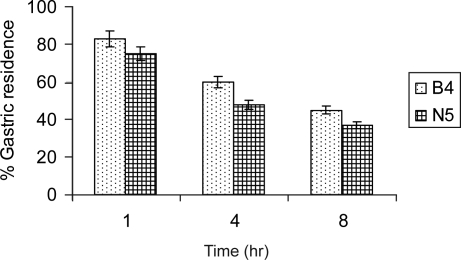
Gastric residence pattern of batch B_4_ and N_5_ in the stomach in different time interval.

Gastric residence studies were carried out to ensure the transit of the designated formulation in the gastric mucosa. Profile of the percentage of the gastric residence of the microbeads is shown in figure 2. Gastric retention of selected batch B and batch N were found 45±1.2% and 37±1.5% at 8 hrs of the study. Significant difference of the gastric retention of batch B, may be due to the affinity of the formulation to glycoproteins of the mucous layer of the stomach. The formulation was floated in the stomach and later adhered to the mucous layer so both mechanisms enhanced the gastric residence time.

#### Evaluation of the concentration of the drug in gastric mucosa

**Figure 3:**
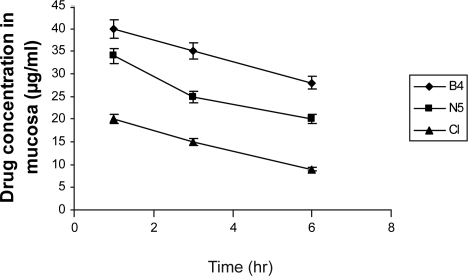
Drug concentration in gastric mucosa of the experimental animal treated with batch B_4_, N_5_ and Cl in different time intervals.

The drug concentration in gastric mucosa of the experimental animal was evaluated in order to assess availability of the drug in the vicinity of mucosal layer in different time period. The pattern of release of Cl from the formulation of batch B_4_, batch N_5_ and plain claritromycine (batch Cl) are shown in figure 3. The drug was released from the formulation and attained initially concentration of 40±0.05 µg/ml (batch B), 34±0.02 µg/ml (batch N) and 20±0.06 µg/ml (batch Cl). Thus, high concentration of the drug release was from the batch B_4_ in to the gastric mucosa because of the partition of the drug from the formulation barrier.

**Table 3:** Characteristics of prepared formulation of polymeric blended gellan gum gel beads

Formulation		Diameter (mm)^a^,^b^		Floating ability (%)^a^		Encapsulation Efficiency (%w/w)^a^,^c^		Drug content%^a^,^c^	
				
COB	HOB	COB	HOB	COB	HOB	COB	HOB	COB	HOB
B_1_	N_1_	1.05±0.8	0.94±0.4	95±1.4	86±1.4	78±0.6	73 ±0.3	66±1.5	54±1.4
B_2_	N_2_	1.10±0.5	1.02±0.5	92±1.6	83±1.6	82±0.4	70 ±0.5	60±1.8	47±1.5
B_3_	N_3_	1.37±0.5	1.08±0.6	87±1.4	78±1.4	75±0.7	72 ±0.5	58±1.4	44±1.6
B_4_	N_4_	1.48±0.3	0.99±0.4	92±1.2	80±1.5	95±0.8	81±0.4	78±1.6	63±1.4
B_5_	N_5_	1.28±0.8	1.30±0.5	84±1.3	89±1.6	93±0.4	86±0.6	70±1.9	68±1.8
B_6_	N_6_	1.40±0.6	1.22±0.6	76±1.4	73±1.5	83±0.5	78±0.4	61±1.7	66±1.5
B_7_	N_7_	1.08±0.2	1.07±0.6	71±1.4	70±1.4	84±0.4	81±0.8	63±1.3	60±1.6
B_8_	N_8_	1.23±0.3	1.20±0.2	79±1.4	73±1.5	81±0.8	79±0.5	62±1.6	58±1.3

COB=gellan-carbopol blended formulation.

HOB=gellen -HPMC blended formulation.

a =Mean±SD (n=3)

b =number of micro beads taken for determination of particle size (n=20)

c =Drug content in each100 mg of bead

#### In Vitro drug release studies

**Figure 4:**
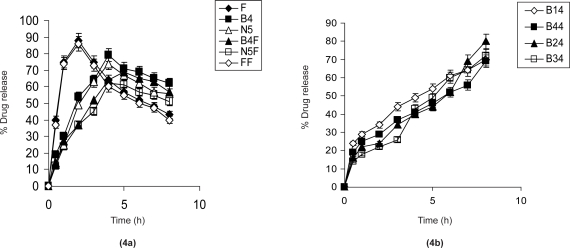
Comparative drug release profile: (4a) fasting and fed state condition (4b) ethyl cellulose coated batch B_4_.

*Study of the* In vitro Cl release of gel beads was carried out both in the SGF solution (fasting state) and in phthalate buffer (fed state) for a period of 8 hrs. The results indicate that 88±1.2% of the pure drug (batch F) was dissolved within 2 hrs in fasting state (pH of 1.2) and in the fed state (batch FF, pH of 3.4), the release was 86.9±1.6%..After 8hrs, drug release from batch B4 was 62.0±2.2% (fasting state) from the batch B F was 57.0±2.5% (fed state) in the SGF and phthalate buffer solution (Fig 4a) respectively. Drug release from the optimized formulations B_4_ followed the Higuchi (R2=0.924) and Peppas models (R2=0.936, n=0.36) and suggested a diffusion based mechanism of the drug release as the diffusion exponent values were less than 0.45^13^–(16). However, the dissolution profiles of all EC- coated beads were best fitted to the zero-order kinetic model (Fig 4B). The batch B_24_which showed highest release (Table 2) of clarithromycin (80±1.6%) was regarded as pH sensitive controlled release formulation of clarithromycin.

## CONCLUSION

The developed optimized formulation is anticipated to maintain minimum inhibitory concentration (MIC) of clarithromycin at the infection site and allowing penetration of the drug inside the mucous gel initially. It is also anticipated that the developed formulation release the drug to the eradicate infection lesion in the acidic region of the gastrointestinal tract. Hence clarithromycin delivery system which was designed, not only could curtail and alleviate the shortcomings of conventional drug delivery vehicles, but also targeted to deliver antimicrobial agents like clarithromycin to the infected *H. Pylori* cell lines.
